# A production platform for disulfide-bonded peptides in the periplasm of *Escherichia coli*

**DOI:** 10.1186/s12934-024-02446-6

**Published:** 2024-06-05

**Authors:** Martin Gibisch, Matthias Müller, Christopher Tauer, Bernd Albrecht, Rainer Hahn, Monika Cserjan-Puschmann, Gerald Striedner

**Affiliations:** 1 Christian Doppler Laboratory for Production of Next-Level Biopharmaceuticals in E. coli, Institute of Bioprocess Science and Engineering, Department of Biotechnology, University of Natural Resources and Life Sciences, Vienna, Muthgasse 18, 1190 Vienna, Austria; 2 Boehringer-Ingelheim RCV GmbH & Co KG, Dr.-Boehringer-Gasse 5-11, Vienna, Austria

**Keywords:** Recombinant peptides, CASPON™ tag, Somatostatin, Aprotinin, Plectasin, Parathyroid hormone, Fed-batch cultivation

## Abstract

**Background:**

Recombinant peptide production in *Escherichia coli* provides a sustainable alternative to environmentally harmful and size-limited chemical synthesis. However, in-vivo production of disulfide-bonded peptides at high yields remains challenging, due to degradation by host proteases/peptidases and the necessity of translocation into the periplasmic space for disulfide bond formation.

**Results:**

In this study, we established an expression system for efficient and soluble production of disulfide-bonded peptides in the periplasm of *E. coli*. We chose model peptides with varying complexity (size, structure, number of disulfide bonds), namely parathyroid hormone 1–84, somatostatin 1–28, plectasin, and bovine pancreatic trypsin inhibitor (aprotinin). All peptides were expressed without and with the N-terminal, low molecular weight CASPON™ tag (4.1 kDa), with the expression cassette being integrated into the host genome. During BioLector™ cultivations at microliter scale, we found that most of our model peptides can only be sufficiently expressed in combination with the CASPON™ tag, otherwise expression was only weak or undetectable on SDS-PAGE. Undesired degradation by host proteases/peptidases was evident even with the CASPON™ tag. Therefore, we investigated whether degradation happened before or after translocation by expressing the peptides in combination with either a co- or post-translational signal sequence. Our results suggest that degradation predominantly happened after the translocation, as degradation fragments appeared to be identical independent of the signal sequence, and expression was not enhanced with the co-translational signal sequence. Lastly, we expressed all CASPON™-tagged peptides in two industry-relevant host strains during C-limited fed-batch cultivations in bioreactors. We found that the process performance was highly dependent on the peptide-host-combination. The titers that were reached varied between 0.6–2.6 g L^−1^, and exceeded previously published data in *E. coli*. Moreover, all peptides were shown by mass spectrometry to be expressed to completion, including full formation of disulfide bonds.

**Conclusion:**

In this work, we demonstrated the potential of the CASPON™ technology as a highly efficient platform for the production of soluble peptides in the periplasm of *E. coli*. The titers we show here are unprecedented whenever parathyroid hormone, somatostatin, plectasin or bovine pancreatic trypsin inhibitor were produced in *E. coli*, thus making our proposed upstream platform favorable over previously published approaches and chemical synthesis.

**Supplementary Information:**

The online version contains supplementary material available at 10.1186/s12934-024-02446-6.

## Introduction

Peptides (proteins < 100 amino acids) have been gaining great attention for many therapeutic applications. With a strongly increasing market interest and already over 50 peptide-associated drugs on the marked, they are considered next-level biopharmaceuticals [1, 2]. Given their small size, peptides have certain advantages over large therapeutic proteins. These include a high potency of action, wide range of targets, low toxicity, fewer side effects, and a low accumulation in tissues [3]. Peptides offer a broad range of applications, most notably cancer therapy, antimicrobial peptides (AMPs), as well as targeted cargo delivery, Alzheimer’s disease, Malaria, and signaling (peptide hormones) [3–6]. Besides pharmaceutical applications, peptides also find use in the food [7] and cosmeceutical industry [8].

Commonly, peptides are either extracted from biological material or synthesized chemically. Both current state-of-the-art methods for peptide production therefore either rely on the availability of biomaterial for extraction or the heavy use of hazardous agents and solvents [9]. Solid phase peptide synthesis (SPPS) is most frequently used, thereby emphasizing the need for more ecological and sustainable methods for peptide production. Despite being a staple in chemical peptide synthesis, SPPS is yield-limited after a certain number of assembled amino acids, thus not feasible for production of relatively large peptides > 50 amino acids [10]. Additionally, the formation of disulfide bonds (DSB) is complicated and challenging [11].

A variety of expression hosts have thus far been successfully used for recombinant peptide production, such as microbes, yeasts, plant cells, and mammalian cells. Especially yeasts such as *Saccharomyces cerevisiae* and *Pichia pastoris* are frequently used, since they can be cultivated at high cell densities (100–200 g L^−1^) and have the ability to secrete peptides into the cultivation medium. The latter is of particular importance, as the risk of proteolytic degradation can be minimized, and downstream costs can be reduced [12–14]. Microbial cells are another widely used class of expression hosts for peptide production and offer a possibility to increase product quantities in view of the steadily increasing market demand and overcome sustainability challenges. Especially *E. coli* represents a convenient expression host for reducing costs and improving product yields, as the organism is fast-growing, easy to manipulate, cost-effective, and a broad variety of production strains are commercially available [15]. Additionally, *E. coli* offers the possibility to express disulfide bond-containing peptides. The periplasm holds an oxidative environment, where reduced cysteine residues (free thiol groups) of translocated proteins/peptides can be oxidized by thiol oxidases (e.g., Dsb family), and disulfide bonds are formed [16].

Two major translocation pathways are commonly used for translocation of heterologous proteins/peptides into the periplasm, namely the post-translational SecB pathway and the co-translational signal recognition particle (SRP) pathway [17]. In the post-translational SecB pathway, the signal sequence is recognized by the chaperone SecB, which also prevents the translocated protein from folding. Protein translocation is further facilitated by SecA and the SecYEG translocation complex under ATP consumption. The co-translational SRP pathway is naturally used for the translocation of inner membrane (IM) proteins. With the SRP pathway, translation by the ribosome and translocation via the SecYEG complex are coupled. In short, when the signal sequence is recognized and bound by SRP, the ribosome is connected to the SecYEG complex (mediated by the filamentous temperature-sensitive protease H, FtsH), and the protein is synthesized directly into the periplasm or inner membrane without ATP consumption [17, 18]. Signal sequences that have been successfully used for periplasmic protein expression, for example, derived from outer membrane protein A (OmpA) and disulfide bond isomerase A (DsbA) [19, 20].

Peptides are known for being prone to degradation by host proteases and peptidases, especially when recombinantly expressed in *E. coli.* The most frequently used approach to circumvent this undesirable effect is by expressing the peptide in combination with N- or C-terminal fusion proteins or tags. A variety of different fusion partners/tags exist, and most comprise solubility-enhancing features and affinity regions for purification [21]. Amongst several others, fusion partners that were successfully used for enhanced protein or peptide expression are glutathione *S*-transferase (GST, 26 kDa), maltose binding protein (MBP, 43 kDa), and N-utilizing substance A (NusA, 55 kDa). In case of peptide expression, fused expression with these tags is rather unintuitive, as they are relatively large and make up to 90% of the synthesized fusion peptide. Alongside these relatively large fusion partners, smaller fusion partners have been used, namely thioredoxin (Trx, 12 kDa) and the small ubiquitin-related modifier (SUMO, 11 kDa) [2, 21]. Fused to mid- to large-sized peptides (~ 3–9 kDa), however, even the latter would still make up 70–80% of the expressed fusion peptide. To further reduce the mass fraction of the fusion partner, peptides can be expressed with the even smaller novel N-terminal CASPON™ tag, which is only 4.1 kDa in size. The CASPON™ tag comprises solubility enhancing features, as well as a His tag for affinity purification. Additionally, the tag contains a recognition site for efficient tag cleavage by a circularly permutated Caspase-2 (CASPON™ enzyme). With this technology, it was shown that an authentic N-terminus can be generated with all 20 canonical amino acids at the P1’ position [22–25].

Four different peptides were chosen to establish an upstream production platform for efficient peptide expression, namely parathyroid hormone 1–84 (PTH), somatostatin 1–28 (SST), plectasin (PLEC), and bovine pancreatic trypsin inhibitor (BPTI, also known as aprotinin). PTH is a peptide required for the regulation of calcium homeostasis and is naturally produced in the thyroid glands. It is 84 amino acids long and mainly consists of α-helices. PTH is 9.4 kDa in size and contains no disulfide bonds [26]. SST, also known as growth hormone inhibiting hormone, inhibits several signaling pathways that involve gastrointestinal, endocrine, exocrine, pancreatic, and pituitary secretions. The cyclic peptide is 28 amino acids long, 3.1 kDa in size and contains one DSB. Inherently, somatostatin 1–28 is cleaved to the 14 amino acid short derivate somatostatin 1–14 [27]. PLEC is an antimicrobial peptide (defensin) that was first isolated from a saprophytic fungus and was shown to act toxic against Gram-positive bacteria. Mature PLEC consists of 40 amino acids and is 4.4 kDa in size. Typical for defensins, PLEC is rich in cysteines and contains three DSBs. Structurally, the peptide consists of an α-helix and two antiparallel β-strands [28]. BPTI is a naturally occurring inhibitor of trypsin and other proteolytic enzymes. It finds use during surgery to inhibit fibrinolysis and subsequently reduce the risk of bleeding. The peptide is 58 amino acids long and 6.5 kDa in size. It consists of a twisted β-hairpin, a C-terminal α-helix, and like PLEC contains three disulfide bonds [29, 30].

In this study, we expressed four industry-relevant disulfide-bonded peptides with varying complexity (size, structure, number of disulfide bonds) in the periplasm of *E. coli*, with the goal to establish an upstream production platform utilizing the N-terminal CASPON™ tag. PTH, SST, PLEC, and BPTI were expressed with and without the tag, demonstrating the need for a fusion tag to enable efficient expression. Moreover, we show that degradation mainly occurred in the periplasm after the translocation by utilizing different signal sequences for either co- or post-translational translocation into the periplasmic space. Lastly, we compared the expression system in two different host strains, namely BL21(DE3) and HMS174(DE3), during bioreactor cultivations, and found that process performance strongly depends on the host-peptide combination. High soluble peptide titers could be achieved, and DSB formation could ultimately be confirmed. We show here that peptides with varying levels of complexity can be efficiently expressed in *E. coli*, making our proposed platform a valuable contribution to sustainable and economically friendly peptide production.

## Results

### Fusion tag is needed for sufficient peptide expression

To investigate whether the peptides PTH, SST, PLEC, and BPTI, are prone to proteolysis when expressed without a fusion tag, we expressed them without tag and with the N-terminal CASPON™ tag. Moreover, it should be evaluated whether the CASPON™ tag holds its solubility- and expression-enhancing features even when expressed in the periplasmic space, as our previous studies focused on cytoplasmic expression. Amino acid sequences of all peptides are given in the supplementary information (Table S1). The peptides were translocated by utilizing the SecB-dependent pathway for translocation, mediated by the OmpA signal sequence (OmpA^SS^). We cultivated all peptide-producing BL21(DE3) strains with the respective gene of interest integrated into the genome. The cultivations were performed at µL-scale using the BioLector™ system with an applied linear feed profile by enzymatic glucose release from a dextran polymer (constant volume). Peptide expression was induced with 0.5 mM IPTG after 14 h. After induction, the production phase lasted for further 10 h. Induction led to strong deviations in growth, however, this was seemingly peptide-dependent and not uniform for all strains (Figure S1).

**Fig. 1:**
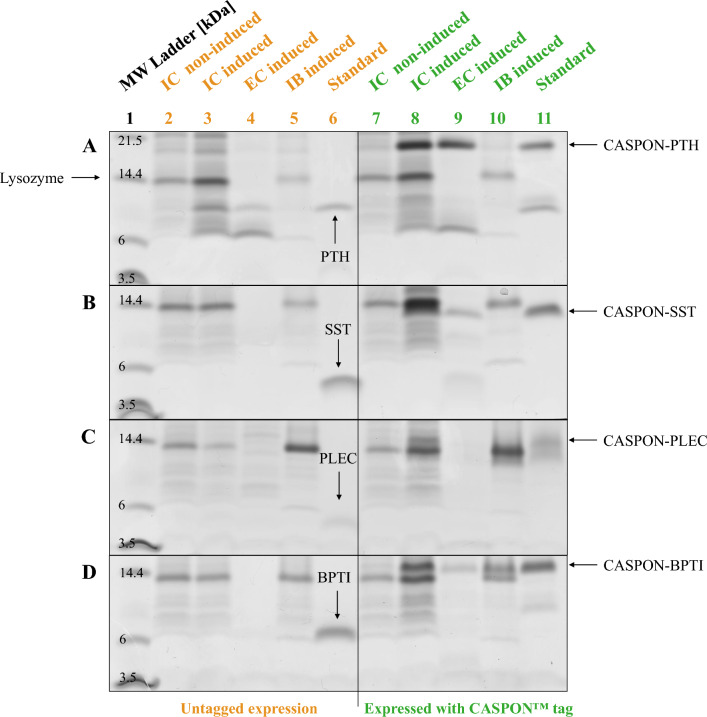
Tricine SDS-PAGE analysis of soluble intracellular (IC), extracellular (EC) and insoluble inclusion body (IB) fractions of peptide-expressing strains. **A** parathyroid hormone 1–84 (PTH), **B** somatostatin 1–28 (SST), **C** plectasin (PLEC), and **D** bovine pancreatic trypsin inhibitor (BPTI), were expressed without tag (lanes 2–6) and fused to the CASPON™ tag (lanes 7–11) during BioLector™ cultivations. Cultures were induced by addition of IPTG to a final concentration of 0.5 mM. Lysozyme used for enzymatic cell lysis is indicated with a black arrow on the side in frame **A** and is represented by the 14.4 kDa mark in lane 1 on all panels. Standards derived from purification of CASPON™-tagged peptides via affinity chromatography and subsequent cleavage of the tag with the CASPON™ enzyme [22], and were added to the respective lanes at 100 mg L^−1^ (plectasin at 50 mg L^−1^). The gels were stained using Coomassie Brilliant Blue, and converted to grey scale after scanning

Tricine SDS-PAGE was used to analyze the peptide contents in soluble intracellular (IC) and extracellular (EC) fractions, as well as in insoluble inclusion bodies (IB). The IC fraction represents the cell lysate after enzymatic cell lysis, while the EC fraction represents the cell-free cultivation medium after centrifugation of the cell suspension. PTH was successfully expressed (Fig. 1A lanes 3–5), however, presumably suffered from severe degradation shown as distinct bands between 6–9 kDa. SST and PLEC were not visible on the gels (Fig. 1B, C, lanes 3–5). BPTI was only very faintly visible in the extracellular fraction (Fig. 1D, lane 4).

**Fig. 2:**
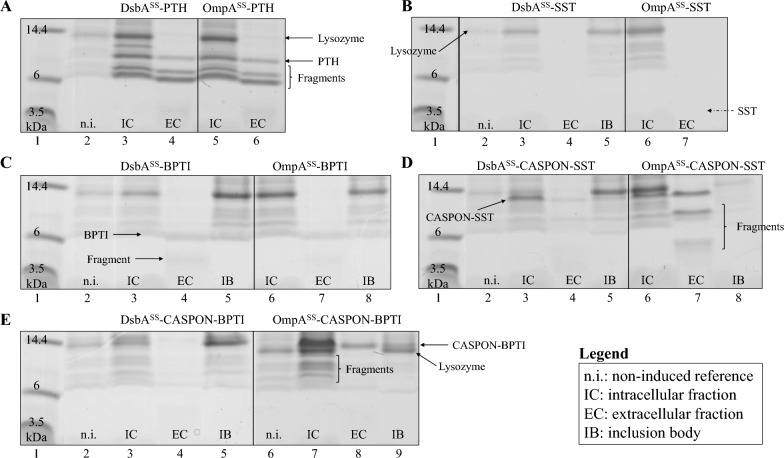
Tricine SDS-PAGE analysis of intracellular (IC), extracellular (EC), and inclusion body (IB) fractions of BL21(DE3) strains expressing peptides fused to different signal sequences. **A** expression of DsbA^SS^-PTH and OmpA^SS^-PTH, **B** expression of DsbA^SS^-SST and OmpA^SS^-SST, **C** expression of DsbA^SS^-BPTI and OmpA^SS^-BPTI, **D** expression of DsbA^SS^-CASPON-SST and OmpA^SS^-CASPON-SST, **E** expression of DsbA^SS^-CASPON-BPTI and OmpA^SS^-CASPON-BPTI. Lysozyme was used for cell lysis and is represented by the 14.4 kDa marker in lane 1 of each panel and indicated by black arrows. The theoretical position (molecular weight) of SST is indicated in **B** as arrow with a dotted line. IC, EC, and IB fractions derived from induced cultures with 0.5 mM IPTG. Non-induced reference cell lysates (intracellular) are indicated as “n.i.”. SS: signal sequence, PTH: parathyroid hormone 1–84, SST: somatostatin 1–28, BPTI: bovine pancreatic trypsin inhibitor (aprotinin), CASPON: CASPON™ tag. Coomassie Brilliant Blue was used for staining. The gels were scanned and subsequently converted to grey scale. The gel shown in **B** derived from a scan that was cropped and edited to place the marker protein mixture (lane 1) next to the IC and EC samples. The non-edited gel is shown in the supplementary material (Figure S2)

In view of the low expression levels when expressed without a tag, peptide production was not efficient. To counteract this, we expressed the peptides fused to the N-terminal CASPON™ tag under the same cultivation conditions. Expression with the tag was greatly enhanced (Fig. 1A–D, lanes 7–10) and all peptides were visible in the soluble fractions (intra- and extracellular) on the SDS-PAGE gels. Bands for the CASPON™-tagged peptides were clearly visible in IC and EC fractions, however, CASPON-PLEC and CASPON-BPTI also formed insoluble inclusion bodies (Fig. 2C, D, lane 10). Notably, peptides fused to the tag appeared to travel shorter distances on the SDS-PAGE gels and were visible above their expected position (e.g., CASPON-PTH at ~ 20 kDa instead of 13.5 kDa, and CASPON-SST at ~ 13 kDa instead of 7.2 kDa). Although expression was enhanced, proteolysis was visible. Especially CASPON-PTH showed distinct degradation bands in intra- and extracellular fractions (Fig. 1A, lanes 8–9) and fragments of CASPON-SST were found in the extracellular fraction below 6 kDa (Fig. 2B, lane 9).

### Peptides are mainly degraded in the periplasmic space

The expression-enhancing CASPON™ tag could not fully prevent proteolysis, as distinct degradation bands were visible in intra- and extracellular fractions. To investigate whether cytosolic or periplasmic proteases are mainly responsible for degradation of our model peptides, we exchanged the post-translational OmpA^SS^ with the co-translational DsbA signal sequence (DsbA^SS^). With this approach, we sought to circumvent cytoplasmic proteases by directly synthesizing the peptides into the periplasmic space. For these experiments, we chose peptides with and without CASPON™ tag that appeared to be impacted the most by proteolysis: PTH, SST, BPTI, CASPON-SST, and CASPON-BPTI. Opposite to the BioLector™ cultivations in the previous section, we expressed the peptides in BL21(DE3) using the pET30a*cer*-system and did not integrate the sequences into the genome of the host. All cultures were induced with 0.5 mM IPTG after 18 h of cultivation and cultivated for 8 further hours before samples were drawn.

Minor restrictions in cell growth compared to the respective uninduced reference cultivations were visible for each strain upon induction (Figure S3). The deviation was more pronounced when peptides were expressed in combination with the OmpA^SS^ (except for CASPON-SST). PTH was expressed at similar levels with both signal sequences according to the respective peptide bands at roughly 9 kDa (Fig. 2A). However, an additional band at ~ 11 kDa was visible when expressing DsbA^SS^-PTH. SST was still not visible when expressed in combination with the co-translational signal sequence (Fig. 2B). Peptide bands containing BPTI were clearly visible in the extracellular fraction at ~ 6 kDa (Fig. 2C, lanes 4 and 7), however, appeared to be of equal thickness independent of signal sequence. Utilization of the DsbA^SS^ resulted in lower expression levels for CASPON-SST and CASPON-BPTI, respectively (Fig. 2D, E). In case of PTH, CASPON-SST, and CASPON-BPTI, peptide degradation was evident since peptide fragments were visible as distinct bands with the same molecular mass for both signal sequences. Interestingly, degradation fragments were mainly visible in the intracellular fraction for CASPON-BPTI, while for CASPON-SST they appeared to be predominantly extracellular. Our results indicate that proteolysis seemingly occurred independent of the respective signal sequence. Therefore, we conclude that proteolysis mainly occurred *after* their translocation by periplasmic and/or outer membrane proteases. To identify the presumed degradation fragments, selected peptide bands that are indicated as “fragments” in Fig. 2 were cut out of the respective gels and analyzed via mass spectrometry (LC–ESI–MS/MS). All cut-out peptide bands contained major segments of the respective peptide (Figure S4). The sequence of BPTI at 4 kDa on the SDS-PAGE gel (Fig. 2C, lanes 4 and 7) was covered at 79% of the whole peptide. CASPON-SST was identified in all analyzed gel bands (Fig. 2D, lane 7, at ~ 4, 8, and 10 kDa) with a coverage of 47, 78, and 92%, respectively. Note that due to altered migration behavior in combination with the strongly charged CASPON™ tag, positions on the SDS-PAGE gel do not reflect the actual molecular weight.

In addition to the peptide fragments that were found during SDS-PAGE analysis in the crude cell lysates, a cleavage site within SST could be identified via mass spectrometry during purification experiments (independent of this study). CASPON-SST was extracted from the cells and further purified. After cleavage with the CASPON™ enzyme for tag removal and incubation with dithiothreitol (DBS reduction), a second peak was visible on the RP-HPLC chromatograms. Subsequently, both peaks were eluted and analyzed via mass spectrometry. Removing the last four amino acids from the full-length SST peptide, it was found that the resulting molecular weight aligns with that of the identified fragment shown by mass spectrometry (Figure S5). We therefore conclude that the peptide fragment resulted from the cleavage between the Thr-Phe bond at the 24th and 25th position.

### Process performance during bioreactor cultivations depends on peptide-host combination

To evaluate the potential of our CASPON™-tagged model peptides for scalable production processes, we performed bioreactor cultivations with two different *E. coli* production hosts of high industrial relevance, namely B strain-derived BL21(DE3) and K-12 strain-derived HMS174(DE3). We chose to carry out these cultivations in DASGIP® bioreactors at benchtop-scale (2.1 L), as our cultivations at microliter-scale did not allow for active regulation of volume, pH, and pO_2_. In view of our previous results, CASPON-PTH, CASPON-SST, CASPON-PLEC, and CASPON-BPTI were expressed with the OmpA^SS^ for translocation into the periplasm. All strains contained the respective expression cassettes integrated into their genomes.

#### Growth kinetics

**Fig. 3:**
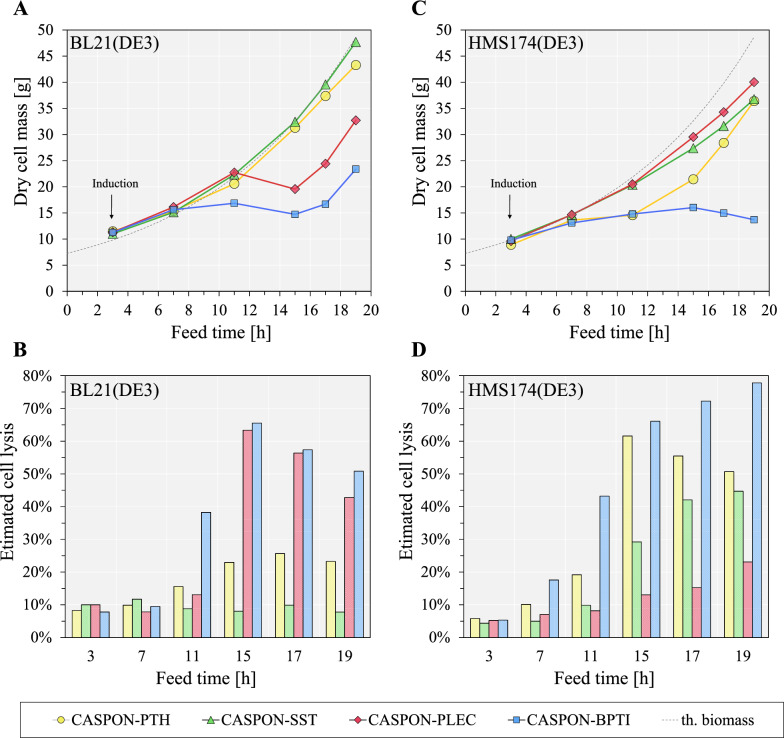
Growth curves and estimated cell lysis of peptide-producing BL21(DE3) (**A**, **B**) and HMS174(DE4) (**C**, **D**) during fed-batch cultivations. Dotted lines represent theoretical growth curves (th. biomass) according to a constant specific growth rate, µ, of 0.1 h^−1^ and theoretical biomass yield, Y_X/S_, of 0.33 and 0.4 for HMS174(DE3) and BL21(DE3), respectively. PTH: parathyroid hormone, SST: somatostatin 1–28, BPTI: bovine pancreatic trypsin inhibitor. Yellow, green, red, or blue markers and bars represent BL21(DE3) or HMS174(DE3) cultures expressing CASPON-PTH, CASPON-SST, CASPON-PLEC, or CASPON-BPTI, respectively

The growth behavior of each strain varied strongly upon IPTG induction, depending on the expressed peptide. For B strain-derived hosts, expression of CASPON-SST or CASPON-PTH did not show a strong influence, whereas CASPON-PLEC and CASPON-BPTI expression led to drastically impaired growth (Fig. 3A). This was complemented by analysis of the extracellular DNA content, an indicator for cell lysis (Fig. 3B). Expression of CASPON-BPTI led to relatively strong lysis up to ~ 38% after 11 h, and the CASPON-PLEC-expressing host lysed strongly up to ~ 64% between 11–15 h of feed. The growth arrest of the cultures expressing CASPON-PLEC and CASPON-BPTI was not maintained, as both strains showed increasing biomass and decreasing levels of cell lysis after 15 h onwards. Similar growth behavior was observed when the peptides were expressed in HMS174(DE3) strains (Fig. 3C). On HMS174(DE3), however, peptide expression apparently had a more drastic impact regarding growth and cell lysis. Expression of CASPON-PTH and CASPON-SST led to stronger deviations from the theoretical growth curve and an even more severe impact could be observed when CASPON-BPTI was expressed. HMS174(DE3)-derived strains predominantly showed higher levels of cell lysis than BL21(DE3)-derived strains (Fig. 3D). On average, cell lysis levels were up to 1.7-, 2.6-, and 1.2-fold higher for expression of CASPON-PTH, CASPON-SST, and CASPON-BPTI, respectively. Contrary to these results, the HMS174(DE3) strain expressing CASPON-PLEC showed a vastly different growth behavior, as this culture reached the highest biomass and lowest cell lysis levels compared to the other HMS174(DE3) strains. Only slight DNA release was detected (between 5–23% of lysed cells). Compared to the respective B strain expressing CASPON-PLEC, cell lysis levels were up to fivefold lower over the time course of the cultivation with the K-12 strain.

#### Peptide expression

**Fig. 4:**
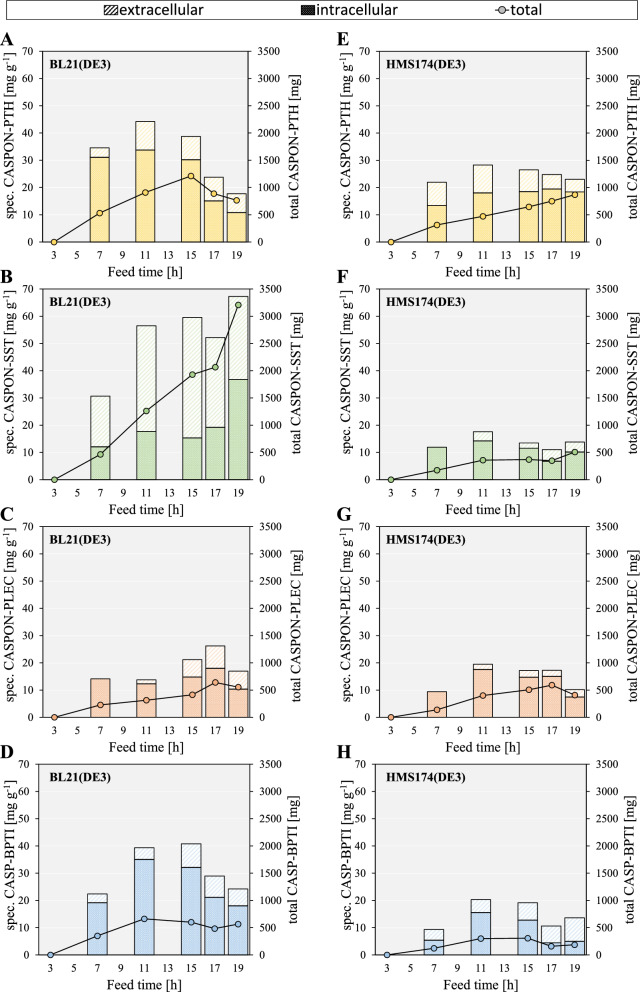
Soluble specific and total peptide titers over the time course of bioreactor cultivations estimated via RP-HPLC analysis. Peptide expression in BL21(DE3) (**A**–**D**), peptide expression in HMS174(DE3) (**E**–**H**). Expression cassettes were integrated into the genome of the respective host strain. CASP: CASPON™ tag, PTH: parathyroid hormone 1–84, SST: somatostatin 1–28, PLEC: plectasin, BPTI: bovine pancreatic trypsin inhibitor (aprotinin)

Soluble IC and EC peptide contents were quantified via RP-HPLC. Inclusion bodies were formed only to a minor extent (Figure S6) and were therefore neglected for analysis. With BL21(DE3)-derived strains, maximum total (IC + EC) specific peptide contents of 44.2, 59.5, 26.2, and 40.8 mg per g DCM of soluble CASPON-PTH, CASPON-SST, CASPON-PLEC, and CASPON-BPTI was produced, respectively (Fig. 4A–D colored bars). These maxima were reached at different timepoints during the cultivations. The specific amount of CASPON-PTH reached its maximum after 11 h, while specific CASPON-PLEC and CASPON-BPTI titers were maximal after 17 and 15 h, respectively. In contrast, CASPON-SST was produced at relatively high rates throughout the whole process and reached its maximum specific amount at the end of the cultivation (19 h feed). To monitor the proteolysis throughout the cultivations, the total peptide amounts for each strain were considered. The maximum total soluble peptide amount (Fig. 4A–D colored dots) was reached at different timepoints in contrast to the total specific peptide content. While CASPON-BPTI production was maximal after 11 h, CASPON-PTH and CASPON-PLEC production reached the individual maximum after 15 and 17 h, respectively. Again, the maximum total amount of CASPON-SST was reached after 19 h, and peptide content steadily increased over time despite strong degradation of the peptide (supplementary material Figure S6B, lanes 12 and 14). In total, 1212 mg (1.2 g L^−1^) CASPON-PTH, 3207 mg (2.6 g L^−1^) CASPON-SST, 639 mg (0.6 g L^−1^) CASPON-PLEC, and 663 mg (0.8 g L^−1^) CASPON-BPTI could be produced at their maximum, respectively. Furthermore, the majority of CASPON-SST was found to be extracellular despite low lysis levels, indicating peptide release out of the periplasmic space independent of cell lysis.

Recombinant peptide production in *E. coli* HMS174(DE3) gave predominantly lower peptide titers in comparison to B strain-derived BL21(DE3), as shown in Fig. 4E–H (colored bars). The maximum total (IC + EC) specific peptide amounts were 28.3, 17.6, 19.5, and 20.3 mg g^1^ for CASPON-PTH, CASPON-SST, CASPON-PLEC, and CASPON-BPTI production, respectively. These maximums were altogether reached after 11 h. Shown in Fig. 4E–H (colored dots), the maximum total amounts were 870 mg (0.7 g L^−1^ after 19 h) CASPON-PTH, 510 mg (0.4 g L^−1^ after 19 h) CASPON-SST, 591 mg (0.5 g L^−1^ after 17 h) CASPON-PLEC, and 307 mg (0.3 g L^−1^ after 15 h) CASPON-BPTI, respectively. In comparison, the total specific amounts reached with the BL21(DE3) strains were 1.3-, 4.0-, 1.3-, and 2.2-fold higher on average. Similar observations were made when comparing the total peptide amounts. On average, peptide production in B strains was roughly 1.5-fold (CASPON-PTH), 4.7-fold (CASPON-SST), 1.1-fold (CASPON-PLEC), and 2.6-fold (CASPON-BPTI) higher compared to K-12 strains, respectively.

#### Degradation by host proteases

Peptide titers appeared to be host-dependent and declined over time for certain peptide-host-combinations. These observations strongly indicate unwanted proteolysis by the hosts, which is supported by SDS-PAGE analysis (supplementary material, Figure S6). The distinct degradation bands found in intra- and extracellular fractions were equal to those shown during BioLector™ cultivations at µL-scale (“Peptides are mainly degraded in the periplasmic space” section, confirmed via MS analysis). In case of CASPON-PTH, degradation fragments were clearly visible in EC fractions at around 9 kDa (Figure S6A, E, lanes 5 and 7). This fragment was again analyzed via mass spectrometry, and major fractions of CASPON-PTH could be identified (Figure S7). As previously described, specific and total CASPON-PTH contents decreased after 11 h of cultivation with BL21(DE3). This, however, was not the case for cultivations with HMS174(DE3). With the HMS174(DE3) strain, the CASPON-PTH content steadily increased over time despite visible degradation in extracellular samples. Proteolysis was therefore more distinct in BL21(DE3), despite this strain showing higher total and specific peptide content on average. After 19 h of cultivation, the final CASPON-PTH content in BL21(DE3) was 12% lower compared to HMS174(DE3). In EC samples containing CASPON-SST (Figure S6B, lanes 12 and 14), severe degradation was visible. Interestingly, although the peptide was obviously degraded, CASPON-SST production in BL21(DE3) showed the highest specific and total (intact) peptide titers among all peptide/host combinations with a steady increase throughout the whole cultivation. In contrast, CASPON-SST production in HMS174(DE3) was drastically lower. Expression of CASPON-PLEC was similar for both production hosts. Both, BL21(DE3) and HMS174(DE3) showed similar specific titers (on average and maximal) and total peptide content curves indicate similar expression/proteolysis activities. Similar to CASPON-SST production, BL21(DE3) was the superior production host for expression of CASPON-BPTI.

### The peptide upstream platform process ensures an authentic N-terminus and complete disulfide bond formation

**Fig. 5:**
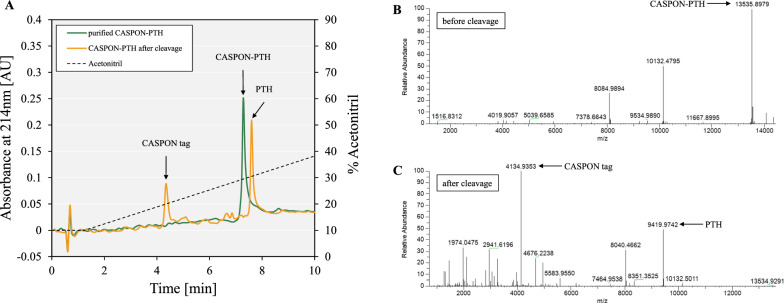
Reversed phase HPLC (**A**) and mass spectrometry (**B**, **C**) analysis of purified CASPON-PTH before and after cleavage of the N-terminal CASPON™ tag by cpCaspase-2. The purified CASPON™-tagged peptide and the peptide mixture after cleavage are represented as the green and yellow lines in the chromatogram, respectively. Eluates were analyzed via RP-LC-ESI-OT-MS. Arrows in the spectrograms indicate the respective peptides. CASPON: CASPON™ tag, PTH: parathyroid hormone 1–84

Peptide production was greatly enhanced when utilizing the N-terminal CASPON™ tag, however, correct expression and successful DSB formation should be confirmed to validate this expression system. For analytical purposes, we purified all peptides by using the previously developed CASPON™ purification platform [22, 23, 25]. RP-HPLC and mass spectrometry analysis were performed before and after cleavage of the tag by the CASPON™ enzyme. Figure 5A exemplarily shows the chromatograms of purified CASPON-PTH before (green line) and after (yellow line) treatment with the CASPON™ enzyme for tag removal. CASPON-PTH eluted after 7.3 min as a single peak. The tag was successfully cleaved, and two distinct peaks were visible after 4.2 (CASPON™ tag) and 7.6 min (PTH), respectively. The chromatograms before and after cleavage of the remaining peptides are shown in the supplementary information (Figure S8A, B, C). Notably, the CASPON™ tag consistently eluted after 4.2 min in all samples. Before cleavage, a distinct peak at 13,535.90 Da was identified (Fig. 5B), which matched the molecular weight of CASPON-PTH as predicted by the ExPASy online tool. After cleavage (Fig. 5C), the CASPON™ tag was identified with 4134.93 Da (4134.94 Da predicted), as well as sole PTH with a molecular weight of 9419.97 Da (9419.97 Da predicted). Chromatograms and spectrograms of analysis with CASPON-SST, CASPON-PLEC, and CASPON-BPTI are given in the supplementary material (Figure S8D-I). Complementary to the results for CASPON-PTH, all peptides (before and after cleavage) were identified with the correct molecular weight with negligible deviation (Table 1). The molecular mass was predicted with the ExPASy online tool PeptideMass [31, 32], already considering hydrogen atom removal due to DSB formation (− 2 Da for SST and − 6 Da for PLEC and BPTI, respectively). The identified and predicted molecular weights matched accurately, indicating complete DSB formation for SST, PLEC, and BPTI. To verify correct DSB formation, commercially available peptide standards of SST and BPTI were compared to the peptides purified via RP-HPLC during this study. The chromatograms are shown in the supplementary material (Figure S9). For both SST and BPTI, the purified and the respective purchased standards eluted at the same time as distinct single peaks. With our method, we only identify single peaks for both the purchased and the purified peptides. The presence of mis-bridged peptide isomers can therefore be ruled out, indicating correctly formed DSBs.

**Table 1:** Mass spectrometry results for identification of peptides before (with CASPON™ tag) and after (without CASPON™ tag) treatment with the CASPON™ enzyme

Peptide	Predicted MW [Da]	Identified MW [Da]
CASPON-PTH	13,535.90	13,535.90
PTH	9419.97	9419.97
CASPON-SST	7263.42	7263.42
SST	3147.49	3147.47
CASPON-PLEC	8515.76	8515.69
PLEC	4399.83	4399.77
CASPON-BPTI	10,624.02	10,623.95
BPTI	6508.10	6509.04
CASPON™ tag	4134.95	4134.95

The molecular weight (MW) of each peptide was determined using the ExPASy online tool PeptideMass. The identified MW of the CASPON™ tag represents the average of the MW identified for each peptide after cleavage. CASPON: CASPON™ tag, PTH: parathyroid hormone 1–84, SST: somatostatin 1–28, PLEC: plectasin, BPTI: bovine pancreatic trypsin inhibitor (aprotinin)

## Discussion

Recombinant peptide production in microbial cells is a more sustainable, economical, and more easily scalable alternative to chemical synthesis, most notably for peptides > 30 amino acids [33]. However, although protein expression in *E. coli* has been vastly studied throughout the last decades, peptide production still remains a major challenge in industrial biotechnology [2]. With our results, we show that *E. coli*, despite the several drawbacks of peptide expression in microbes, is a relevant production host.

In our study, we developed an upstream production platform for the periplasmic expression of different peptides that varied in size, structure, and number of disulfide bonds. The peptide titers that were achieved in this work exceeded those of previously published studies. We expressed four genome-integrated peptides, PTH, SST, PLEC and BPTI, without a tag and with the N-terminal CASPON™ tag. Previous studies with the CASPON™ tag were focused on cytoplasmic protein expression [22, 23]. Here, we sought to gain insight whether the tag still holds its positive influence on solubility and expression enhancement even when the tagged peptides are expressed in the periplasm. As most untagged peptides are poorly expressed in host cells due to proteolytic degradation by intracellular proteases and peptidases, we also wanted to draw a comparison between tagged and untagged peptide expression to investigate whether our model peptides are prone to degradation. Without the tag, recombinant peptide production was not successful. Only PTH and, to a very low extent, BPTI were expressed, while SST and PLEC were not detectable (Fig. 1, lanes 3–5). In combination with the short CASPON™ tag (4.1 kDa), all four peptides could be expressed and were clearly visible on Tricine SDS-PAGE gels as distinct bands (Fig. 1, lanes 8–10).

Peptides, especially when recombinantly expressed in *E. coli,* are highly susceptible to degradation by host proteases. Naturally, proteases inactivate short-lived regulatory proteins and degrade unwanted or incorrectly synthesized proteins [34]. Degradation is usually initiated by large AAA^+^ proteases (ATPases associated with diverse cellular activities) that consist of several subunits and unfold and cleave target proteins into smaller fragments under ATP consumption. This ATP-dependent step is a “try and try again” approach that also serves as control for substrate specificity, usually for larger proteins. After unfolding and initial cleavage, the protein fragments are further degraded into smaller fragments and eventually into single amino acids by various endo- and aminopeptidases [34–36]. Due to their small size, most peptides do not undergo the ATP-consuming control mechanisms and are therefore more likely to be degraded directly by various proteases and aminopeptidases in an ATP-independent matter [37]. Peptide fragments resulting from degradation were still visible even when fused to the CASPON™ tag, thereby demonstrating that proteolysis was not completely prevented. Proteases that could be involved in the degradation are the periplasmic Protease III (PtrA) or the membrane protease LoiP (also known as YggG, protease domain exposed to periplasm), both known for cleavage between Phe-Phe bonds (e.g., present in SST) and unspecific cleavage of peptides smaller than ~ 7 kDa [38, 39]. Moreover, due to the strong negatively charged and solubility enhancing features of the T7AC element of the CASPON™ tag [23], degradation by proteases that preferentially degrade hydrophobic proteins/peptides or protein/peptide aggregates might be reduced. Among others, these can include cytoplasmic proteases (in case of post-translational translocation with the OmpA^SS^) like ATP-dependent ClpAP or HslUV [37, 40], and periplasmic proteases like DegP, DegQ, or TSP [41–43]. A cleavage site including a Phe residue was identified at the 24Thr-Phe25 bond in SST. However, the cleavage appeared only after purification and treatment with dithiothreitol (reduced DSB). It is therefore difficult to pinpoint a specific protease, as cytoplasmic and periplasmic proteases might have been present after cell lysis. Moreover, this suggests that the presence of correctly formed DSBs reduces the susceptibility to proteolysis, likely because the potential cleavage sites is shielded and not accessible for the protease/peptidase. Numerous cyto- and periplasmic proteases and aminopeptidases exist in *E. coli*, and many cleave proteins/peptides in an unspecific manner [34, 37, 44]. It is therefore challenging to predict which exact proteases or peptidases are responsible for the cleavage of our model peptides (or any protein/peptide in general).

We expressed the most severely degraded peptides in combination with the co-translational DsbA^SS^. The goal was to investigate whether our model peptides are predominantly degraded in the cytosol before their translocation or in the periplasm after their translocation. Peptide expression with the DsbA^SS^ resulted in higher biomass generation throughout the cultivations, compared to peptide expression with the OmpA^SS^ (Figure S3). Tricine SDS-PAGE revealed that this is likely due to reduced peptide expression with the DsbA^SS^ and therefore lower imposed metabolic burden [45]. With the co-translational signal sequence, bands ascribed to the respective peptides were considerably thinner (Fig. 2). Translocation with the DsbA^SS^-mediated SRP pathway is ATP-independent; thus, the translocation efficiency (translocated amino acids per second) is roughly ten times lower compared to the ATP-dependent SecB pathway [46]. Moreover, the strong T7 system rapidly depletes resources for host mRNAs synthesis by its tremendous transcription rate. In consequence, the efficiency of the SRP pathway might be reduced even further due to the requirement of 4.5S RNA for translocation [17, 47]. When expressing PTH in combination with the DsbA^SS^, an additional band at roughly 11 kDa was visible on the respective SDS-PAGE gel (Fig. 2A). No additional bands were visible when expressing CASPON-SST, CASPON-BPTI, or BPTI in combination with the DsbA^SS^. This could imply insufficient cleavage of the signal sequence, which appears to be peptide-dependent. A similar effect during the translocation of different host proteins was reported before, thereby strengthening this hypothesis [48]. As the degradation bands found on SDS-PAGE gels and identified via mass spectrometry were indistinguishable for both signal sequences, we conclude that degradation appeared to be mostly mediated by periplasmic proteases.

Most *E. coli* strain derivatives cannot or only poorly secrete recombinant proteins into the medium [49]. The product remains intracellular, unless the outer membrane is permeabilized by external influences or due to directed mutagenesis in the host genome. Interestingly, some of the analyzed peptides or identified degradation fragments thereof were found in both intra- and extracellular fractions although no severe cell lysis was observed, depending on the peptide. For instance, fragments of BPTI and CASPON-SST were found exclusively in the extracellular fractions, while CASPON-BPTI fragments were only found in intracellular fractions, and PTH fragments were found in both fractions (Figs. 1, 2). We therefore assume that certain peptides or fragments were released from the periplasm by passive transport during the cultivations. Peptide analysis via RP-HPLC of intra- and extracellular fractions from the bioreactor cultivations strengthens this assumption, as a large fraction of intact CASPON-SST was found in the extracellular space although cell lysis levels were low. This phenomenon is a poorly understood process. It is generally believed that protein export to the external environment in *E. coli* is limited to pathogenic strains [50]. However, it was found that the protein YebF encoded by both pathogenic and nonpathogenic strains of *E. coli* is destined to the extracellular medium, suggesting that also non-pathogenic strains could harbor pathways to secrete certain proteins [51]. If released passively (or by a to date uncharacterized pathway), this would also imply that peptides could be degraded by outer membrane proteases, such as OmpT (in case of *E. coli* K-12 derivatives) [52] or its close relative OmpP [53]. Moreover, BL21(DE3) produced higher amounts of extracellular peptides compared to HMS174(D3) independent of lysis (in case of CASPON-SST expression). Again, this might indicate passive transport due to high periplasmic peptide contents and the ease of leakage due to the small size. This is a probable result of different outer membrane compositions, as BL21(DE3) lacks the outer membrane proteins OmpC and ButB [54], hinting that these proteins might influence the outer membrane permeability.

The most successful expression system (OmpA^SS^-CASPON-Peptide) was compared in two different host strains, *E. coli* BL21(DE3) and HMS174(DE3), during bioreactor cultivations. We found that process performance was dependent on the combination of peptide and host (Fig. 3). Both strains showed severe cases of cell lysis, especially when CASPON-BPTI was expressed (independent of titers), while lysis levels were lower when CASPON-SST was expressed. Cell lysis appeared to be depending on the expressed peptide, however, in case of CASPON-PLEC also depended on the host strain. Expression of CASPON-PLEC led to 65% of lysed cells in BL21(DE3) cultures, however, only ~ 25% lysed during HMS174(DE3) cultures (Fig. 3B, D). In addition, CASPON-PTH production was less affected by proteolysis when produced in HMS174(DE3), shown by steadily increasing total product titers (Fig. 4E). This could be a result of the lower susceptibility to stress [54, 55] and potentially lower stress-related protease activity in the K-12 strain. To the contrary, HMS174(DE3) was found to lyse more strongly compared to BL21(DE3) when expressing antibody fragments [20]. Taking this into account, the production host needs to be selected even more carefully with the goal to maximize the product yields of different classes of biopharmaceuticals.

After the bioreactor cultivations, the produced peptides were extracted, purified, and analyzed via mass spectrometry before and after cleavage with the CASPON™ enzyme (Fig. 5). The molecular mass that was identified for the peptides before and after cleavage matched the calculated molecular weight as predicted (Table 1), including hydrogen removal after DSB formation. The occurrence of disulfide-bonded mis-bridged peptide isomers was shown before [56, 57]. Therefore, to exclude the presence of mis-bridged isomers, we compared purchased SST and BPTI standards to our own purified standards via RP-HPLC (Figure S9). Both the purchased and our own purified standards eluted at the same time as single peaks. The reduction of DSBs was previously shown to result in altered hydrophobicity of the affected peptide [58]. Thus, mis-bridged peptide isomers could be ruled out, as the separation technique used in this study was based on hydrophobicity, and only single peaks were observed. We therefore conclude that, (1) the produced peptides contained correctly formed DSB, and (2) that the CASPON™ tag was cleaved off completely, leaving behind an authentic N-terminus. This confirms previously published data with large proteins (9–50 kDa) [24], affirming that the CASPON™ platform can also be applied to mid- to large-sized peptides (3–9 kDa). It has to be noted that, in this study, no activity assays were performed, as this was beyond the frame of our study. These experiments including the purification and implementation of activity assays will be published within a follow-up study.

PTH, SST, PLEC, and BPTI have been subject to several studies before and were expressed in combination with various fusion proteins or tags. Recombinant human PTH has been produced in the cytosol of *E. coli* mostly as fusion protein with TrxA up to 1300 mg L^−1^ [59–61], but also with the CASPON™ tag up to 1.5 g L^−1^ [23]. Both derivates of SST (1–14 and 1–28) could be produced as fusion peptides in combination with a β-galactosidase fragment, TrxA, and a combination of different fusion peptides up to 300 mg L^−1^ [62–65]. Recombinant PLEC was produced in *E. coli* as fusion peptide in combination with TrxA, GST, and SUMO during shake flask cultivations up to 35–92 mg L^−1^ [66–70]. Lastly, BPTI was expressed as fusion peptide (fused to alkaline phosphatase) or as concatemer. However, to date, yields in *E. coli* were poor (6 mg L^−1^), and higher titers (426 mg L^−1^) were only achieved when using eukaryotic organisms like *Saccharomyces cerevisiae* [71–75]. When utilizing the CASPON™ tag for periplasmic peptide production in combination with the OmpA-derived signal sequence, we reached titers that exceed previously published results 8.7-fold for tagged SST, 6.7-fold for tagged PLEC, and 133-fold for tagged BPTI (1.9-fold when compared to expression in *S. cerevisiae*). Our reported maximal CASPON-PTH titer reached about 90% of the maximal published titer. However, here the tagged PTH was expressed in the periplasm whereas in previous studies it was expressed in the cytoplasm. The improvement when using the CASPON™ technology for peptide expression becomes especially evident when the molecular weight of the tag and the resulting mass fraction of the peptide are considered. The untagged peptide titers were improved 1.3-fold for PTH, 22-fold for SST, 15-fold for PLEC, and 625-fold for BPTI. An overview is shown in Table 2. However, it has to be emphasized that, besides *E. coli*, other organisms are also commonly used for recombinant peptide production. These organisms include other microbes such as *Bacillus subtilis*, yeasts like *S. cerevisiae* or *P. pastoris*, as well as plant cells, insect cells or mammalian cells. In particular, organisms with high secretion efficiency such as *P. pastoris*, where the resulting follow-up costs can be easily minimized, are often used [12–14]. Each organism harbors certain advantages and disadvantages. It is therefore important to carefully evaluate the most-suited expression host, depending on the desired peptide, production scale, post-translational modifications (either desired or unwanted), downstream applications, and process costs. Considering the variety of production hosts, a direct comparison between *E. coli* and other host would comprise a global evaluation of all factors that influence the overall manufacturability. Consequently, we focus on *E. coli* for our comparison.

**Table 2:** Comparison between peptide titers shown in this study and previously published data

	This study		Literature		Fold increase	
	Tagged [g L^−1^]	Untagged [g L^−1^]	Tagged [g L^−1^]	Untagged [g L^−1^]	Tagged	Untagged
PTH	1.2	0.8	1.3 [59]*	0.6	0.9x	1.3x
SST	2.6	1.1	0.3 [64]	0.05	8.7x	22x
PLEC	0.6	0.3	0.09 [66]*	0.02	6.7x	15x
BPTI	0.8	0.5	6 * 10^–3^ [73]*	0.8 * 10^–3^	133x	625x

Volumetric titers of this study represent the maximal titers reached during bioreactor cultivations (“Peptide expression” section)

Data taken from literature represent the maximal published titer. Comparisons were drawn from yields of the respective peptides expressed in *E. coli*. Titers of untagged peptides derived from calculating the mass fraction of the peptide from the respective fusion peptide. References of titers taken from literature are given in brackets behind the titer of tagged peptide. Peptide titers from references marked with * derived from peptide expression during shake flask cultivations

Another important aspect that has to be considered is that, after extraction and purification, the final concentrations, as shown in our study, will be lower. The titers in our study were analyzed directly after cell lysis with HCl, thereby not reflecting the titers after downstream processing. Studies regarding the optimization of purification methods for CASPON™-tagged peptides are currently ongoing, but were beyond the frame of this study. Lastly, it has to be noted that for scaled-up industrial production processes, further optimization would be necessary to fully exploit the positive effect of the CASPON™ tag on expression while simultaneously minimize cell lysis. In case of PTH, SST, PLEC, and BPTI expression in *E. coli*, the titers shown here are already promising, especially since our production processes were not optimized and were served exclusively for screening. With our results, we place *E. coli* as a valuable system for recombinant peptide expression and further enhance the toolset of the organism, making it a relevant contribution to research and industry.

## Conclusion

In the present study, we demonstrate that recombinant peptides fused to the CASPON™ tag can be expressed at high levels in the periplasmic space of *E. coli* with complete DSB formation and an authentic N-terminus after tag removal. Our proposed expression platform was successfully applied to four different model peptides of varying complexity, expressed in two different production hosts. We believe that our proposed platform for peptide production in *E. coli* serves as valuable alternative to chemical synthesis and is an impactful contribution to research and industry. However, it has to be pointed out that despite high titers were reached, degradation was still clearly evident. Depending on the peptide, degradation fragments can make up a large fraction of the total peptide content. At this end, optimization efforts regarding host/peptide combination, tackling proteolysis, and production process optimization need be implemented to further unlock the potential of recombinant peptide expression in *E. coli* for the future.

## Material and methods

### Strains and plasmids

**Fig. 6:**
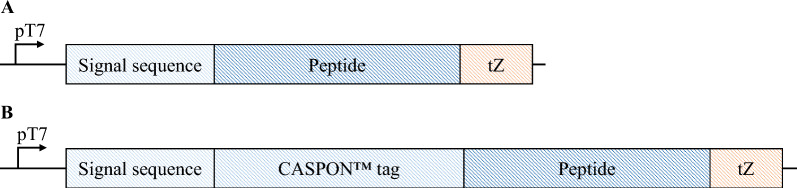
Schematic overview of cassettes for periplasmic peptide expression without tag (**A**) or in combination with the N-terminal CASPON™ tag (**B**). All expression cassettes contained the T7 promoter, as well as the tZenit terminator [76]. Peptides were expressed with either the OmpA signal sequence (OmpA^SS^) or the DsbA signal sequence (DsbA^SS^). Note that size proportions in the figure do not reflect the actual length of each sequence

PTH, SST, PLEC, and BPTI were expressed in the periplasm of *E. coli*. For translocation, the peptides were expressed in combination with signal sequences either derived from outer membrane protein A (OmpA) or disulfide bond isomerase A (DsbA). The peptides were either expressed without tag (mature form) or fused to the N-terminal CASPON™ tag (4.13 kDa) [22, 23]. The expression cassettes are schematically shown in Fig. 6. Transcription was stopped by the synthetic tZenit terminator element [76]. Expression cassettes were either integrated into the host genome of BL21(DE3) (New England Biolabs, USA) and HMS174(DE3) (Merck Millipore, Germany) via a previously described method [77] or cloned into a pET30a plasmid backbone containing a *cer* [78] sequence (pET30a*cer*) with subsequent transformation into the production hosts. Amino acid sequences of all signal sequences and peptides are listed in the supplementary material (Table S1).

### Media

Precultures for all cultivations were carried out in semisynthetic medium (SSM), which per liter contained 3 g KH_2_PO_4_, 4.58 g K_2_HPO_4_, 0.1 g tryptone, 0.05 g yeast extract, 0.25 g tri-sodium citrate, 0.1 g MgSO_4_ * 7H_2_O, 0.01 g CaCl_2_ * 2H_2_O 150 µL trace element solution (see below), 0.45 g (NH_4_)_2_SO_4_, 0.37 g NH_4_Cl, and 10 g glucose monohydrate. Trace element solution contained FeSO_4_ * 7H_2_O (40 g L^−1^), MnSO_4_ * H_2_O (10 g L^−1^), AlCl_3_ * 6H_2_O (10 g L^−1^), CoCl_2_ * 7H_2_O (7.3 g L^−1^), ZnSO_4_ * 7H_2_O (2 g L^−1^), Na_2_MoO_4_ * 2H_2_O (2 g L^−1^), CuCl_2_ * 2H_2_O (1 g L^−1^), and H_3_PO_3_ (0.5 g L^−1^).

Cultivations in BioLector™ were carried out in medium that per liter contained 5 g glucose monohydrate, 33 g EnPump200 dextran (EnPresso, Germany), 0.6% 3000 U mL^−1^ amylase enzyme (EnPresso Reagent A, EnPresso, Germany) 31.04 g 3-(*N*-morpholino)-propane sulfonic acid (MOPS), 7.41 g (NH_4_)_2_SO_4_, 2.22 g K_2_HPO_4_, 2.22 g tri-sodium citrate, 1.48 g Na_2_SO_4_. 0.74 g NH_4_Cl, 7.41 mg thiamine hydrochloride, 0.37 g MgSO_4_ * 7H_2_O, and 0.74% v v^−1^ trace element solution. Trace element solution for BioLector™ medium contained ZnSO_4_ * 7H_2_O (4.07 * 10^−4^ g L^−1^), CuSO_4_ * 5H_2_O (3.7 * 10^−4^ g L^−1^), MnSO4 * H2O (2.22 * 10^−4^ g L^−1^), FeCl_3_ * 6H_2_O (3.09 * 10^−2^ g L^−1^) EDTA (2.47 * 10^−2^ g L^−1^), CoCl_2_ * 6H_2_O (4.07 * 10^−2^ g L^−1^) and CaCl_2_ * 2H_2_O (1.48 * 10^−3^ g L^−1^).

Bioreactor cultivations were carried out as fed-batch cultivations. Therefore, batch and feed media were prepared separately. Batch medium contained per liter 6.27 g KH_2_PO_4_, 2.12 g 85% H_3_PO_4_, 1.5 g yeast extract, 2.74 g tri-sodium citrate, 0.46 g MgCl_2_ * 6 H_2_O, 0.2 g CaCl_2_, 0.5 mL trace element solution (see above), 3.02 g (NH_4_)_2_SO_4_, and 33 g glucose monohydrate. Fed-batch medium contained per liter 2.44 g MgCl_2_ * 6H_2_O, 1.07 g CaCl_2_ * 2H_2_O, 2.65 mL trace element solution, and 174.64 g glucose monohydrate.

### Cultivations

Precultures for all processes were grown in shake flasks containing SSM at 37 °C while shaking on an orbital shaker at 180 rpm for ~ 6 h. Kanamycin was added to the medium at 50 µg mL^−1^, whenever the strains carried a pET30a*cer*-derived plasmid to avoid premature plasmid loss.

The BioLector™ system was used for screening experiments at small scale. 750 µL of medium were inoculated with 50 µL of preculture. Cultivations were performed in MTP-48-B FlowerPlates (m2p-labs, Germany) at 30 °C while shaking at 1400 rpm and a humidity of 85%. The cultures were induced by addition of Isopropyl β-d-1-thiogalactopyranoside (IPTG) to a final concentration of 0.5 mM after 14 h. Production phase lasted for 8 more hours before the process was ended and samples were drawn.

For bioreactor cultivations, DASGIP® SR1500ODLS benchtop bioreactors (Eppendorf AG, Germany) were used with the respective hardware for online monitoring and control. During the process, ammonia was used to maintain a neutral pH. Bioreactors were inoculated with 7 mg of biomass (25 OD units of 25 mL preculture). A feed with a specific growth rate, µ, of 0.1 h^−1^ was initiated after the end of batch and maintained for 19 h. After 3 h of feed, the cultures were induced by pulsed addition of 60.2 µmol IPTG (94.4 µM at induction timepoint). During batch phase, the temperature was held at 37 °C and subsequently reduced to 30 °C during fed-batch.

Samples for off-line analysis were drawn throughout the cultivations. These included 1 mL cell suspension for determination of dry cell mass (DCM), 1 mg DCM samples for further cell lysis and analysis of intracellular (IC) peptide/protein content, 30 mg dry cell mass for peptide extraction and HPLC analysis, and cell-free cultivation broth for determination of extracellular (EC) peptide and DNA content. Biomass samples for SDS-PAGE (1 mg) and HPLC (30 mg) analysis were drawn according to an optical density (OD_600_) to biomass concentration ratio of 3.5.

### Analytics

#### Enzymatic cell lysis and SDS-PAGE

Samples containing roughly 1 mg of DCM were drawn throughout the cultivations and were analyzed for intracellular soluble and insoluble peptide content. For this, enzymatic cell lysis was performed. The pellets were resuspended in buffer containing 30 mM Tris/HCl, pH = 8.2, 25 mM EDTA, 10 mM MgCl_2_, 0.8 v v^−1^ NuPage® Reducing Agent (Invitrogen, USA). Then, 50 µL of lysozyme (2 mg mL^−1^ stock) and 50 µL Benzonase (50 U mL^−1^ stock) solution were added and incubated for 10 min while shaking. After incubation, 100 µL of 6% Triton X-100 was added to the mixture and incubated for 10 further minutes while shaking. The mixture was then centrifuged for 10 min at 15,000 rcf and 4 °C. The supernatant (containing the soluble fraction) was transferred to a fresh tube, while the pellet (insoluble fraction) was washed twice with buffer containing 100 mM Tris/HCl (pH = 8.2). Further, the washed pellet was resolved in 400 µL resolving buffer (100 mM Tris/HCl, pH = 8.2, 8 M Urea, 14.3 mM Reducing Agent) and incubated for 30 min while shaking, and subsequently centrifuged as before. After centrifugation, the supernatant contained the resolved insoluble fraction of previous lysis steps (inclusion bodies). Both soluble and insoluble fraction were frozen to − 20 °C until further use.

Soluble and insoluble fractions derived from cell lysis, as well as extracellular samples derived from cell-free cultivation broth after centrifugation were analyzed via Tricine SDS-PAGE [79]. Novex® Tricine 10–20% mini-gels, respective hardware and buffers were used for analysis (purchased from ThermoFisher Scientific, USA). Directly after the SDS-PAGE, the gels were placed into fixing solution (50% v v^−1^ EtOH, 10% v v^−1^ acetic acid) for 20 min. Then, the gels were stained using Coomassie Brilliant Blue staining solution (1.4 mM Coomassie Brilliant Blue R250, 25% v v^−1^ EtOH, 8% v v^−1^ acetic acid) for 30 min. After staining, the gels were de-stained two times in de-staining solution (25% v v^−1^ EtOH, 8% v v^−1^ acetic acid) for 20 min and RO-H_2_O overnight. The gels were scanned, and the scans were converted to grey scale.

#### Extraction with HCl and RP-HPLC

Samples for the soluble intracellular peptides (30 mg pellets) were treated with HCl to extract the acid-stable recombinant peptides and precipitate the majority of host proteins before RP-HPLC analysis. Each cell pellet was resuspended in 2% HCl (in 50 mM Bis/Tris and 300 mM NaCl, pH 8.5) to a concentration of 35 g L^−1^ and incubated for 3 h at room temperature while shaking. Afterwards, the pH was neutralized with 5 M NaOH. The suspension was then centrifuged for 10 min at 15,000 rcf, and the supernatant was transferred to a fresh tube. All samples were frozen to − 20 °C until further use. Before analysis, samples were thawed and filtered through a 0.22 µm filter. For HPLC analysis, a Waters e2695 HPLC (Waters, USA) in combination with a TSKgel Super-Octyl (2.3 µm, 4.6 × 100 mm) reversed phase column (Tosoh Bioscience LLC, USA) was used. Samples were separated with a HQ-H_2_O/Acetonitrile gradient. For separation, the column was heated to 50 °C, and a flow rate of 2 mL min^−1^ was implemented. Peptide peaks were quantified using a standard curve of the respective peptide and a concentration range of 0.125–2.0 g L^−1^.

Peptide standards were prepared after harvesting BL21(DE3) cultures that produced the respective CASPON™-tagged peptides making use of the CASPON™ platform technology for purification [22, 24]. After extraction, the peptides were captured and eluted via an IMAC Nickel Sepharose™ Fast Flow (Cytiva, USA) column. Imidazole was used as the eluting agent and was removed with 3000 MWCO Amicon® Ultrafilters (Merck, USA) by several concentration and dilution steps. The peptide concentration was subsequently adjusted to 1 g L^−1^ with PBS buffer (pH = 7.4). For tag removal, the purified peptide standards were incubated with CASPON™ enzyme at a molar enzyme/peptide ratio of 1:10 for 12 h at room temperature.

#### DNA analysis

Cell lysis was estimated by analysis of DNA content in the cell-free supernatant after centrifugation of cultivation broth. For this, it was assumed that DNA in the cell-free supernatant represents dead cells [19, 80] and can be used to approximate cell lysis. The percentage of lysed cells was calculated assuming a specific DNA content of 20.3 mg per g dry cell mass [81]. The Qubit 4 fluorometer (Thermo Fisher Scientific, USA) and respective reagents were used. Cell lysis was estimated as followed:

1 $$Lysed\;biomass \left[ \frac{g}{L} \right] = \frac{{\left[ {DNA} \right]_{extracellular} }}{{20.3 \frac{mg\;DNA}{{g\;DCM}}}}$$

2 $$Estimated\;cell \;lyis \left[ \% \right] = \frac{Lysed\;biomass}{{Intact\;biomass + Lysed\;biomass}}$$

#### Mass spectrometry

Samples analyzed via mass spectrometry (MS) were either purified via RP-HPLC (in solution) or cut out of SDS-PAGE gels. When purified peptide samples were analyzed, they were separated on a nanoEase C18 column using 0.1% formic acid as aqueous solvent and an acetonitrile gradient elution over 12 min. An Orbitrap MS (Exploris 480, Thermo, USA) equipped with a standard H-ESI source in positive ion mode and DDA mode (data-dependent aquistion; MS/MS for eluting peaks) were used for detection. The instrument was calibrated using Pierce FlexMix Calibration solution (ThermoScientific, USA). Data was visualized using the FreeStyle™ software 1.8 SP1 (Version 1.8.51.0, ThermoScientific).

Cut-out samples were digested in-gel. The proteins were S-alkylated with iodoacetamide and digested with Trypsin (Promega, USA). Digested samples were loaded on a nanoEase C18 column (nanoEase M/Z HSS T3 Column, 100 Å, 1.8 μm, 300 μm × 150 mm, Waters, USA) using 0.1% formic acid (FA) as the aqueous solvent. A gradient from 1% B (B: 80% Acetonitrile, 0.1% FA) to 40% B in 50 min was applied, followed by a 10 min gradient from 40% B to 95% B that facilitates elution of large peptides, at a flow rate of 6 μL/min. Detection was performed with an Orbitap MS (Exploris 480, Thermo, USA) equipped with the standard H-ESI source in positive ion, DDA mode (switching to MS/MS mode for eluting peaks). MS-scans were recorded (range: 350–1200 Da) and the 20 highest peaks were selected for fragmentation. Instrument calibration was performed using Pierce FlexMix Calibration Solution (ThermoScientific, USA).

### Supplementary Information

Supplementary Material 1.

## Data Availability

Not applicable.
