# Supplementary material for: A production platform for disulfide-bonded peptides in the periplasm of Escherichia coli
Source: Microb Cell Fact. 2024 Jun 5;23:166. doi: 10.1186/s12934-024-02446-6 (PMC11155123; doi:10.1186/s12934-024-02446-6)
Supplement: Supplementary file 1 — Supplementary Material 1. [file 12934_2024_2446_MOESM1_ESM.pdf]

A production platform for disulfide-bonded peptides in the periplasm of *Escherichia coli*

Authors: Martin Gibisch[1], Matthias Müller[1], Christopher Tauer[1], Bernd Albrecht[2], Rainer Hahn[1], Monika Cserjan-Puschmann[1,*], Gerald Striedner[1]

Affiliations:

1) Christian Doppler Laboratory for production of next-level biopharmaceuticals in *E. coli*, Institute of Bioprocess Science and Engineering, Department of Biotechnology, University of Natural Resources and Life Sciences, Vienna, Muthgasse 18, 1190 Vienna, Austria

2) Boehringer-Ingelheim RCV GmbH & Co KG, Dr.-Boehringer-Gasse 5-11, Vienna, Austria

*: Corresponding author

Keywords: recombinant peptides, CASPON™ tag, somatostatin, aprotinin, plectasin, parathyroid hormone, fed-batch cultivation

Supplementary material

Table S1: Amino acid sequences of recombinant peptides and signal sequences used in this study. Molecular weight was determined using the ExPASy online tool, assuming complete disulfide bond formation. OmpA: outer membrane protein A, DsbA: disulfide bond isomerase A, SS: signal sequence, PTH: Parathyroid Hormone 1-84, SST: Somatostatin 1-28, PLEC: Plectasin, BPTI: Bovine Pancreatic Trypsin Inhibitor (Aprotinin), MW: molecular weight.

Peptide	Amino acid sequence	MW [Da]	DSB
OmpA^{SS}	MKKTAIAIAVALAGFATVAQA	2046.18	-
DsbA^{SS}	MKKIWLALAGLVLAFSASA	1990.16	-
CASPON™ tag	LEDPERNKERKEAELQAQTAEQHHHHHHGSGVDVAD	4134.95	-
PTH	SVSEIQLMHNLGKHLNSMERVEWLRKKLQDVHNFVALGAPLAPRD AGSQRPRKKEDNVLVESHEKSLGEADKADVNVLTKAKSQ	9419.97	-
SST	SANSNPAMAPRERKAGCKNFFWKTFTSC	3147.45	1
PLEC	GFGCNGPWDEDDMQCHNHCKSIKGYKGGYCAKGGFVCKCY	4399.77	3
BPTI	RPDFCLEPPYTGPCKARIIRYFYNAKAGLCQTFVYGGCRAKRNNFK SAEDCMRTCGGA	6509.04	3

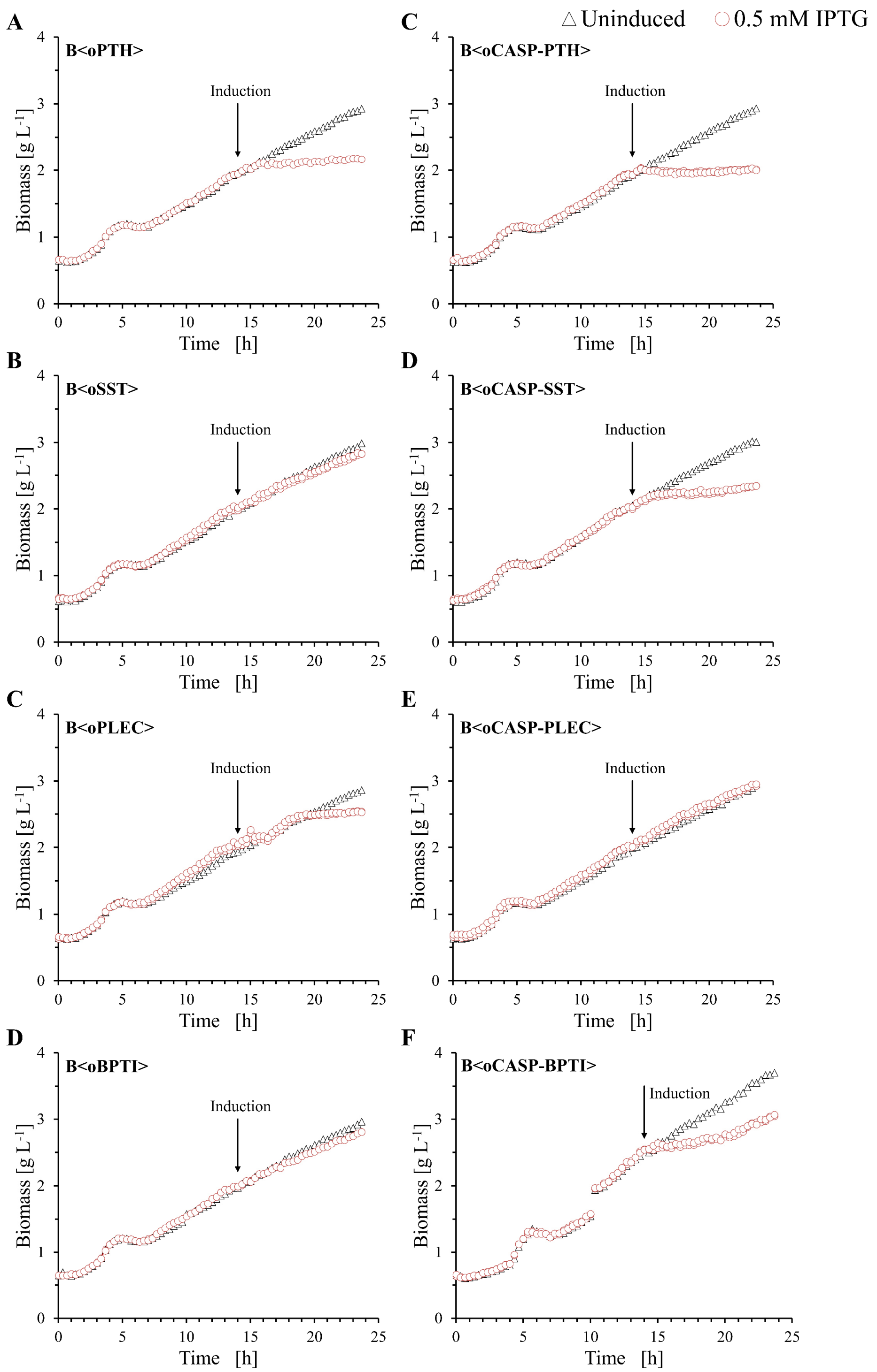

Figure S1: Growth curves of peptide-producing BL21(DE3) strains during BioLector™ cultivations. Cultures were induced with 0.5 mM IPTG (red circles) after 14 hours. Induction is indicated by black arrows. Uninduced reference cultivations are shown as black triangles. PTH: Parathyroid Hormone 1-84, SST: Somatostatin 1-28, PLEC: Plectasin, BPTI: Bovine Pancreatic Trypsin Inhibitor, CASP: CASPON™ tag, o: OmpA signal sequence, < >: genomic integration of the peptide sequence into the host genome. Note that an unknown error occurred during cultivation with B<oCASP-BPTI> that led to shifted biomass curves after roughly 12 hours. The visible bump after 5 hours can be attributed to the complete consumption of batch glucose and subsequent metabolic shift towards residual acetate before further growth under C-limited conditions (after ~7 hours).

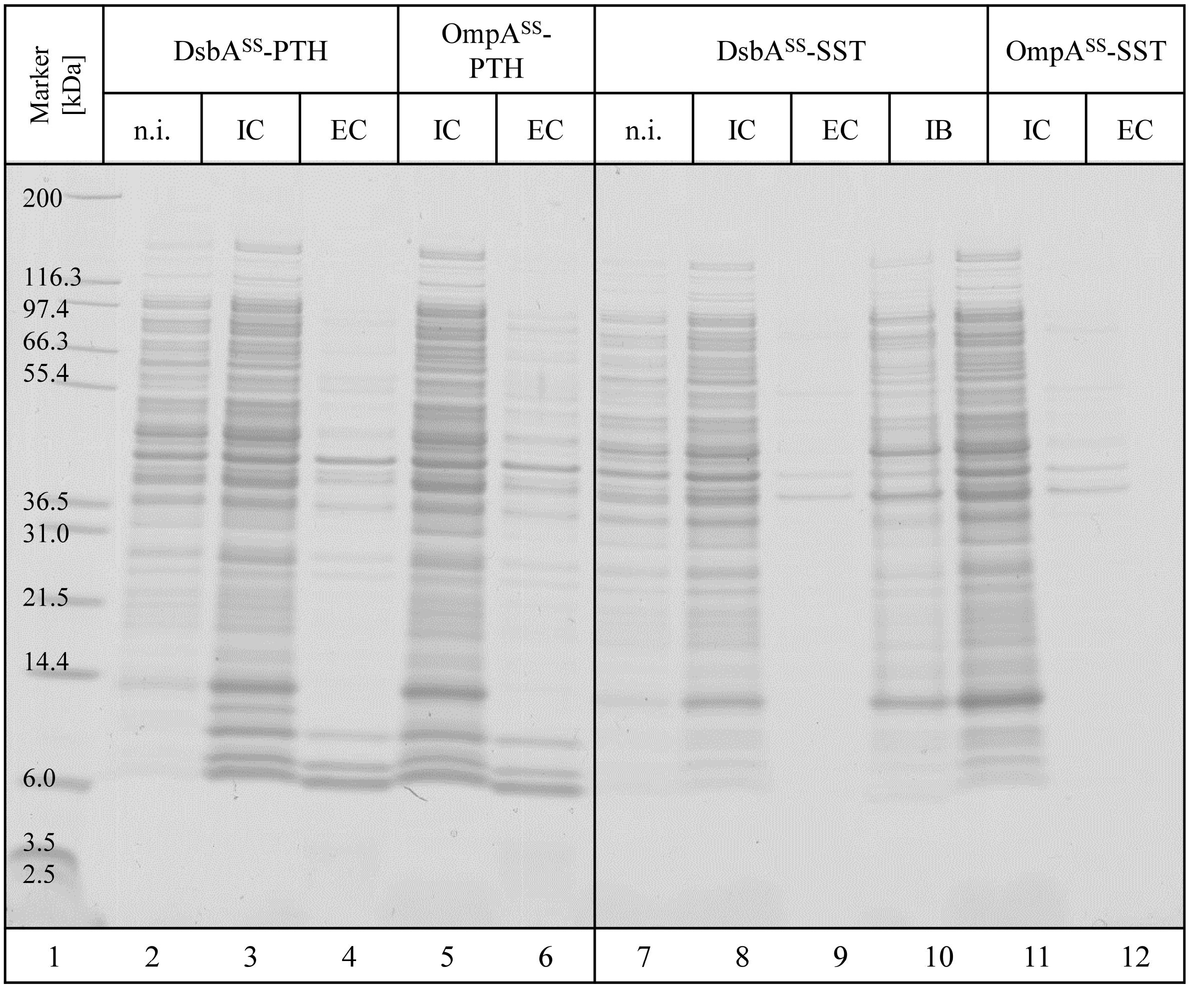

Figure S2: SDS-PAGE analysis of BL21(DE3)-derived strains expressing PTH and SST translocated into the periplasmic space either with the DsbA- or OmpA- derived signal sequences. The band at 14.4 kDa represents lysozyme used for enzymatic cell lysis. n.i.: uninduced intracellular reference sample; IC: intracellular; EC: extracellular; IB: inclusion body; PTH: parathyroid hormone 1-84; SST: somatostatin 1-28.

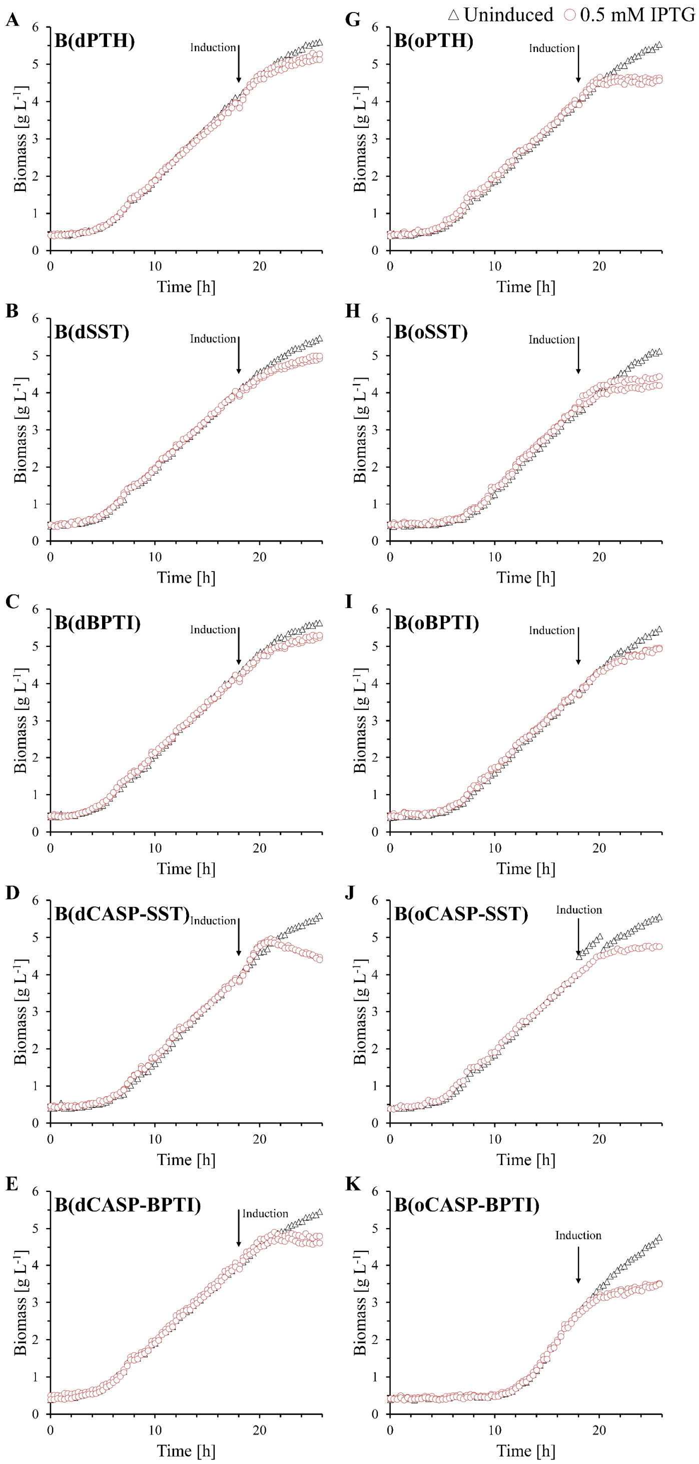

Figure S3: Growth curves of BL21(DE3) cultures producing peptides with different signal sequences. Peptides were either expressed with the DsbA signal sequence or the OmpA signal sequence via the pET30a*cer* expression system. Cultures were induced with 0.01 (blue) and 0.5 mM IPTG (red) after 18 hours of cultivation, respectively. Black triangles represent uninduced reference cultures. B: BL21(DE3), ( ): peptide expression cassette is located on a plasmid, d: DsbA signal sequence, o: OmpA signal sequence, CASP: CASPON™ tag, PTH: Parathyroid Hormone 1-84, SST: Somatostatin 1-28, BPTI: Bovine Pancreatic Trypsin Inhibitor. Note that the prolonged lag phase of B(oCASP-BPTI) was due to insufficient growth during preculture and subsequent lower inoculation biomass.

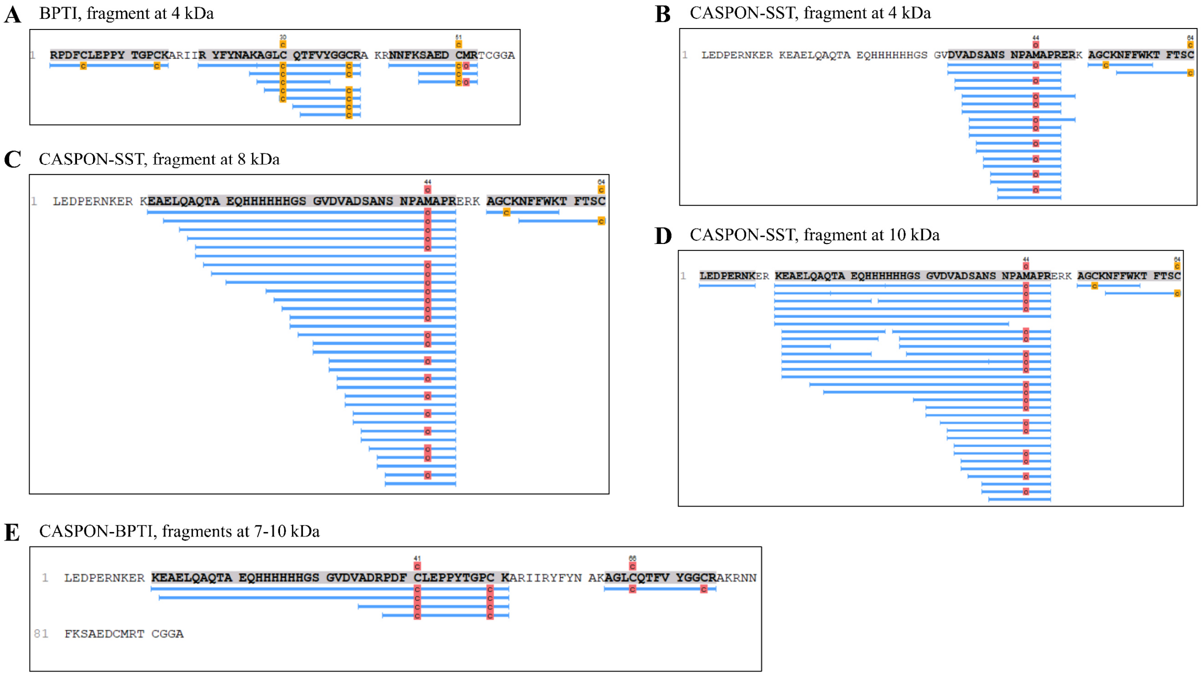

Figure S4: Mass spectrometry (LC-ESI-MS/MS) analysis of degradation fragments found via SDS-PAGE analysis of **A)** BPTI fragment found at 4 kDa on the gel, **B)** CASPON-SST fragments found at 4 kDa on the gel, **C)** CASPON-SST fragments found at 8 kDa on the gel, **D)** CASPON-SST fragment found at 12 kDa on the gel, **E)** CASPON-BPTI fragments found between 7-10 kDa on the gel. Segments highlighted in grey were identified. Blue bars indicate single peptide segments that could be identified. Red and yellow highlights represent oxidation or carbamidomethylation of the respective amino acid. CASPON: CASPON™ tag, BPTI: Bovine Pancreatic Trypsin Inhibitor (Aprotinin), SST: Somatostatin 1-28.

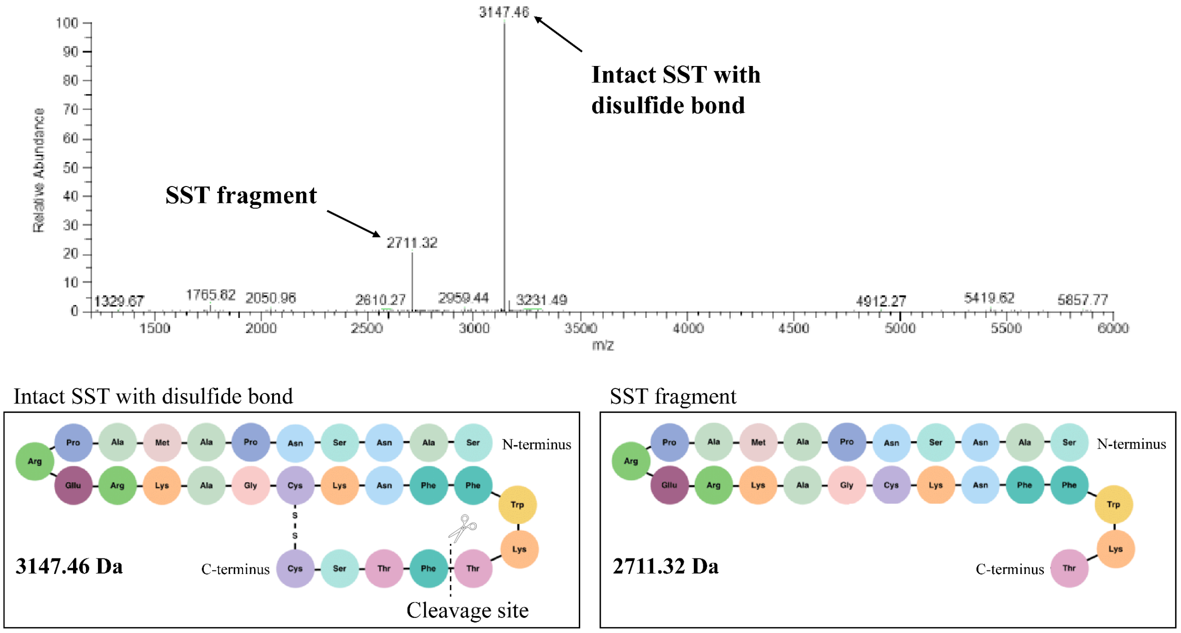

Figure S5: Mass spectrometry analysis of somatostatin 1-28 (SST) before and after cleavage at the Thr-Phe bond. Cleavage appeared after purification and tag removal, when the sample was treated with 5 mM DTT. Images of the amino acid sequence were adapted from (Rogoza et al., 2022).

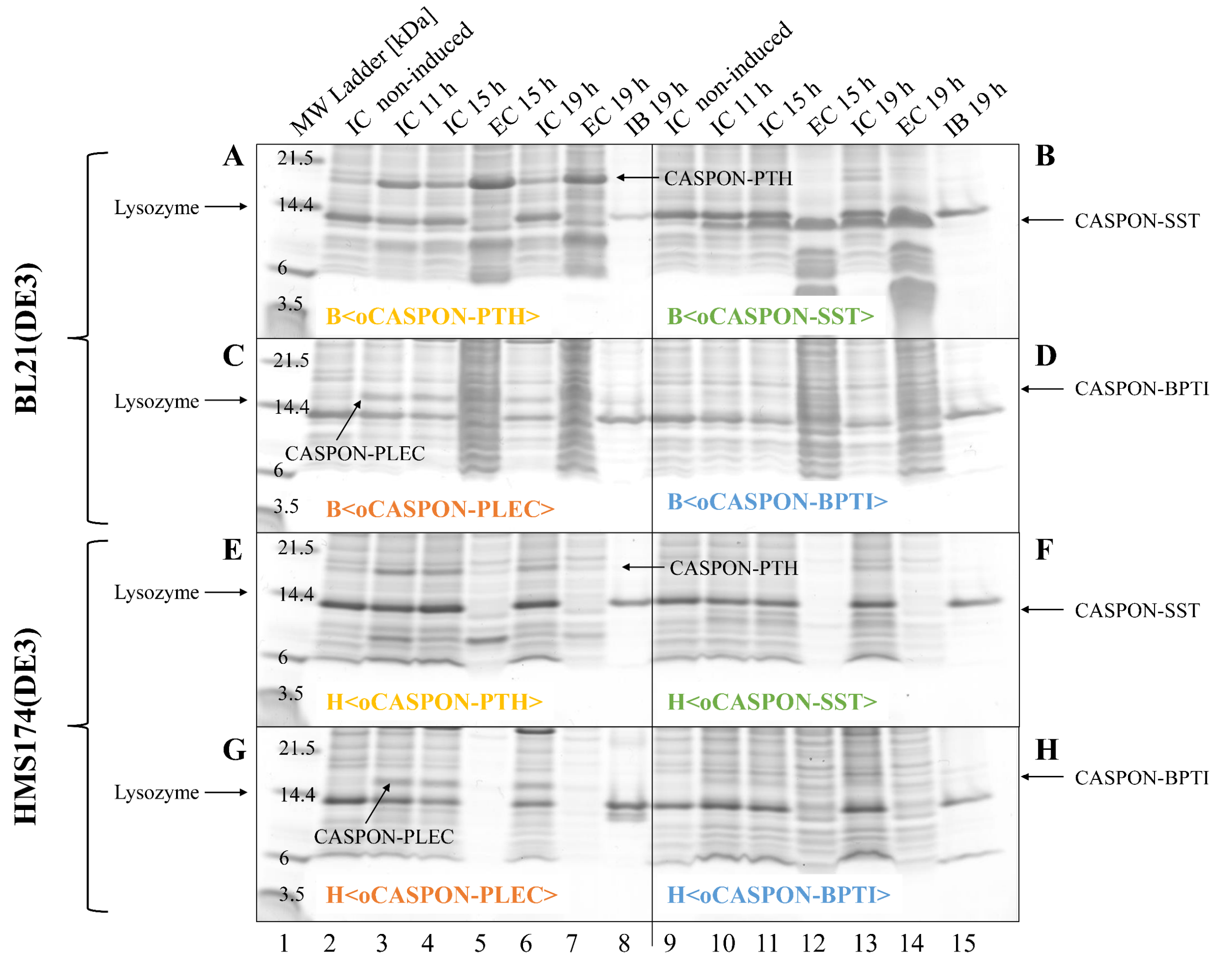

Figure S6: Tricine SDS-PAGE analysis of bioreactor cultivations for recombinant peptide production in BL21(DE3) and HMS174(DE3). **A)** cultivation of B<oCASPON-PTH>, **B)** cultivation of B<oCASPON-SST>, **C)** cultivation of B<oCASPON-PLEC>, **D)** cultivation of B<oCASPON-BPTI>, **E)** cultivation of H<oCASPON-PTH>, **F)** cultivation of H<oCASPON-SST>, **G)** cultivation of H<oCASPON-PLEC>, **H)** cultivation of H<oCASPON-BPTI>. B: BL21(DE3), H: HMS174(DE3), < >: expression cassette is integrated into the genome of the host, o: OmpA signa sequence, PTH: parathyroid hormone 1-84, SST: somatostatin 1-28, PLEC: plectasin. BPTI: bovine pancreatic trypsin inhibitor (aprotinin), IC: intracellular, EC: extracellular, IB; inclusion body. Lysozyme used for cell lysis is indicated as black arrow and the 14.4 kDa marker in the molecular ladder. Note that EC samples (lanes 5, 7, 12, 14) were diluted 1:10 for panels E-H, however, were loaded undiluted for panels A-D.

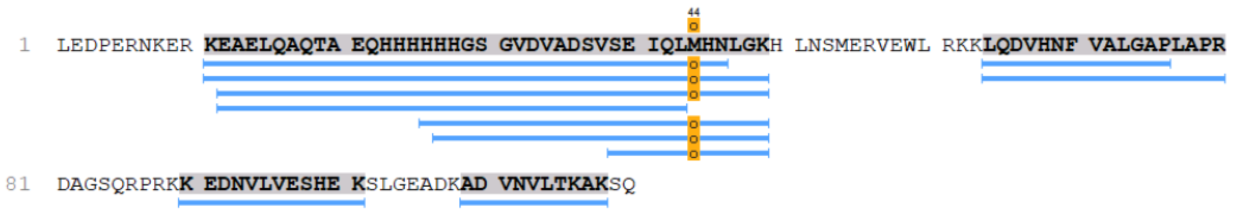

Figure S7: Mass spectrometry analysis of CASPON-PTH fragment found at 9 kDa at extracellular fractions of strains expression CASPON-PTH. Sequences highlighted in grey were identified. Blue segments indicate identified peptide stretches. Yellow highlight indicates oxidation of the respective amino acid. CASPON: CASPON™ tag, PTH: parathyroid hormone 1-84.

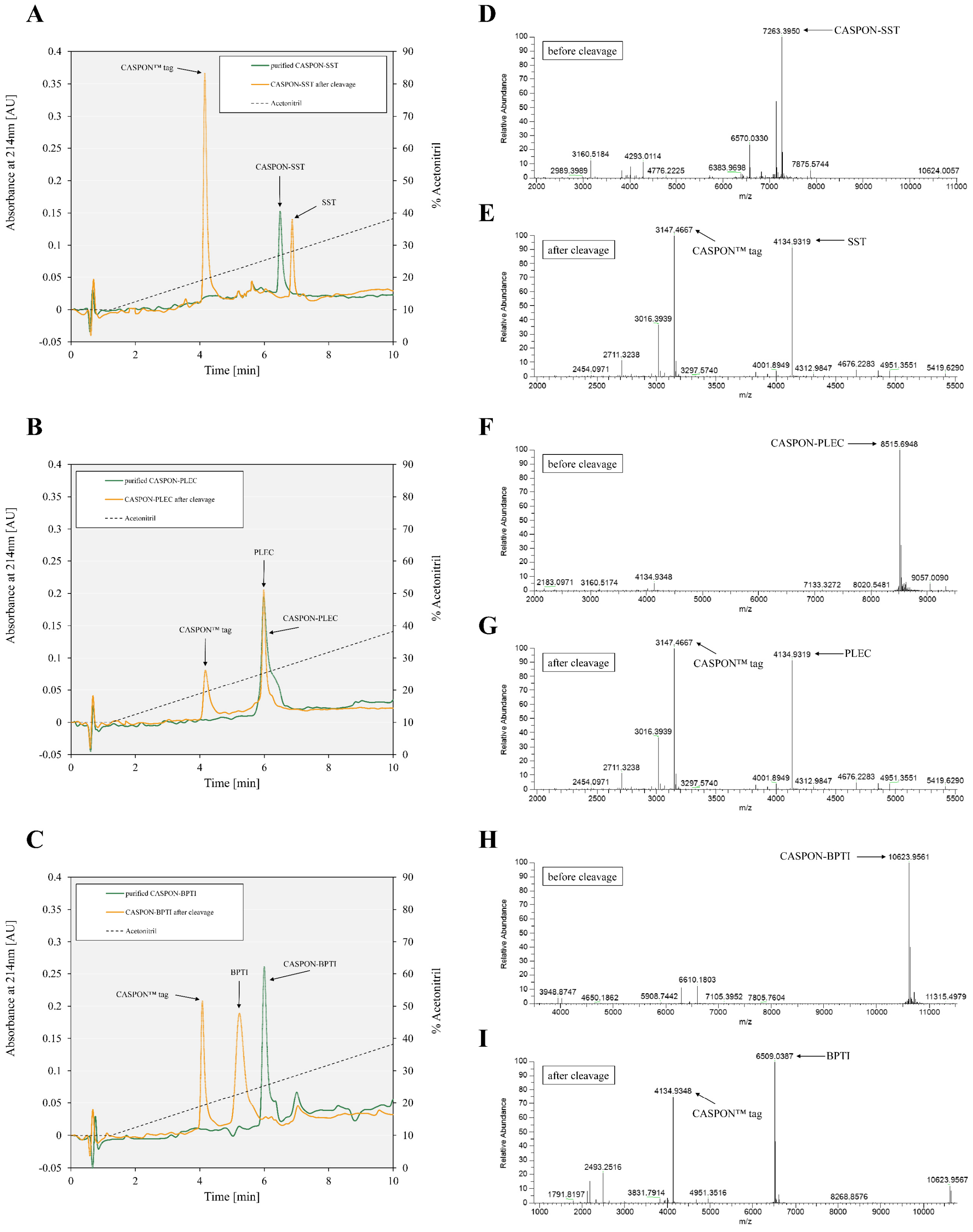

Figure S8: Reversed phase HPLC (**A-C**) and mass spectrometry analysis (**D-I**) analysis of purified peptides before and after cleavage of the N-terminal CASPON™ tag. Arrows in chromatograms and spectrograms indicate the respective peptides. The molecular weights (in Da) of respective peptide peaks are indicated as numbers above the peak.

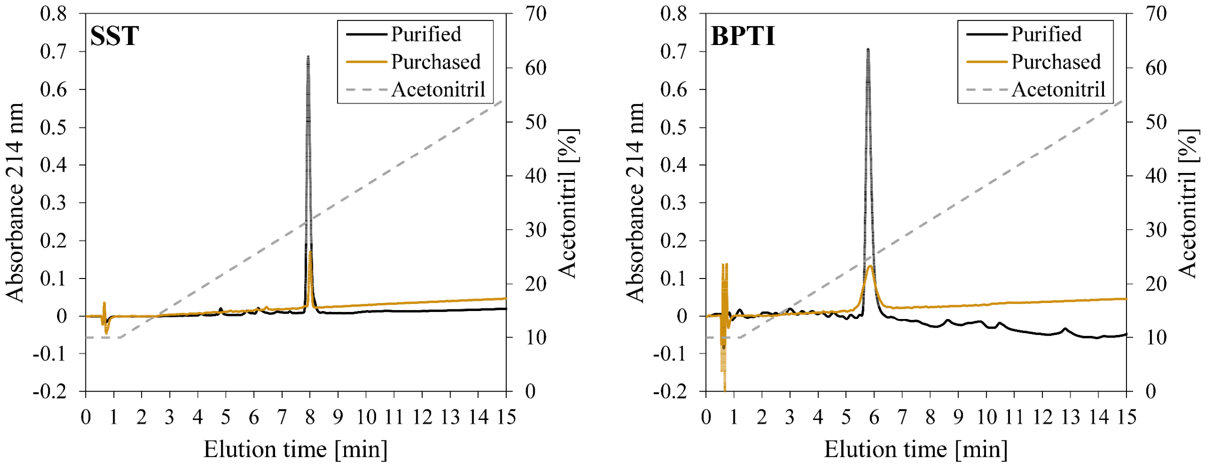

Figure S9: RP-HPLC analysis of purchased (yellow lines) and purified (black lines) peptide standards. The CASPON™ technology was used for purification, and was cleaved off afterwards by treatment with the CASPON™ enzyme. SST: somatostatin 1-28; BPTI: bovine pancreatic trypsin inhibitor (aprotinin).
